# The use of oral recombinant feline interferon omega in two cats with type II diabetes mellitus and concurrent feline chronic gingivostomatitis syndrome

**DOI:** 10.1186/2046-0481-66-19

**Published:** 2013-10-23

**Authors:** Rodolfo O Leal, Solange Gil, Maria TV Brito, David McGahie, Maria MRE Niza, Luís Tavares

**Affiliations:** 1 Centro de Investigação Interdisciplinar em Sanidade Animal (CIISA), Faculdade de Medicina Veterinária, Technical University of Lisbon (TULisbon), Av. Universidade Técnica, 1300-477 Lisbon, Portugal; 2 Virbac, 13e rue LID – BP 27 F, 06511 Carros cedex, France

**Keywords:** Interferon-Omega, Feline, Diabetes, Gingivostomatitis

## Abstract

Feline Chronic Gingivostomatitis Syndrome (FCGS) is a common disease in clinical practice. Among the therapeutic options available, long-acting corticosteroids are frequently used due to their anti-inflammatory and immunosuppressive properties. Although they may improve the clinical symptoms, they can lead to a progressive form of the disease that becomes refractory to treatment. Furthermore, their direct relationship with type II diabetes mellitus (DM) is well known. Consequently, these drugs are controversial and not recommended for routine management of FCGS. Recombinant feline interferon-omega (rFeIFN-ω) is an immunomodulatory compound. Recently, its daily oral administration has been shown to be successful in treating refractory cases of FCGS. This case study describes two clinical cases of type II DM complicated by FCGS. Both animals were calicivirus positive and they had been previously treated with long-acting corticosteroids, which may have been the major cause of DM. The two cats were treated with glargine insulin (Lantus, starting dose 1 IU/cat twice daily (BID)), achieving remission 10 and 18 weeks later respectively. Considering the difficulty with control of FCGS in these animals, an oral daily dose of rFeIFN-ω was started as an alternative to long-acting corticosteroids. In both cats oral clinical signs gradually improved and 60 days after the start of therapy the owners reported a significant relief of pain during mastication. According to the authors’ knowledge, this is the first case report that describes the successful use of rFeIFN-ω in the management of FCGS in type II diabetic cats, in which long-acting corticosteroids are contraindicated.

## Background

Feline chronic gingivostomatitis syndrome (FCGS) is a multifactorial disease, very commonly seen in clinical practice [1]. It is described as a severe oral inflammation (gingivitis, stomatitis and/or periodontitis) and may be secondary to various causes such as neoplasia, toxins or even metabolic disease [2]. In some cases, a cause is not found but regarding the chronic inflammation observed in histopathology samples, an immune mediated etiology should always be considered [3]. Infectious viral diseases are also an important trigger of FCGS [1,4]. Feline immunodeficiency virus (FIV) and feline leukemia virus (FeLV) may lead to FCGS due to an induced immune suppression and dysregulation [5,6]. Additionally feline herpesvirus (FHV-1) and feline calicivirus (FCV) have been well-described as potential factors in the development of FCGS [7]. In fact, a previous study showed that 88% of cats with chronic gingivostomatitis were excreting FHV-1 and FCV [8]. Animals with FCGS usually present with a poor body condition, dysphagia and mild to moderate anorexia [2]. Most therapeutic approaches are not very effective and relapses are frequent [2]. Among the available therapeutic options, dental extraction, antibiotics and corticosteroids or non-steroidal anti-inflammatory drugs (NSAIDs) are usually recommended [2,9]. Due to the doubtful efficacy of NSAIDs over the medium term, long-acting corticosteroids are more frequently used in first-opinion clinical practice [2,3,9]. They are historically described as a good therapeutic approach to FCGS due to the fact that they reduce oral inflammation and control immune-mediated causes, leading to a rapid improvement, an increased appetite and a relief of oral pain [2,3,9]. In spite of their short-term efficacy at controlling the symptoms of the syndrome, long-acting corticosteroids are also a well-known cause of insulin-resistance [10,11], by inducing chronic hyperglycemia which can lead to a glucotoxic beta-cell insufficiency [10]. Therefore, being a potential cause of type II diabetes mellitus (DM) in cats, long-acting corticosteroid use is controversial in this species in general.

In refractory cases of FCGS, where dental extraction and antibiotics are not sufficient to induce remission of the lesions, there are few therapeutic alternatives to corticosteroids [12]. Recombinant feline interferon-omega (rFeIFN-ω; Virbagen, Virbac) is an immune-modulator drug currently licensed in Europe for treatment of feline retroviral infections [13-15]. According to the manufacturer’s instructions, the licensed protocol consists of three cycles of five daily administrations of 1 MU/kg subcutaneously (SC) at days 0, 14 and 60. However other dosages and routes have also been used in the management of other diseases. For instance, alternative subcutaneous and topical protocols were tried in feline coronavirus and FHV-1 infection, respectively [16,17]. Also in cases of FCGS (some of them FCV positive), a study suggested the benefit of intra-lesional administration of rFeIFN-ω [18]. More recently, its oral administration was documented in noneffusive feline infectious peritonitis [19] and in refractory FCGS, particularly in an efficacy study which compared the use of an oral rFeIFN-ω protocol with the use of oral corticosteroids [12]. This study concluded that oral rFeIFN-ω was associated with a significant clinical improvement of FCGS lesions [12]. Furthermore, there was no difference between this protocol and corticosteroids except on pain control, where animals treated with rFeIFN-ω achieved a better pain relief [12]. Therefore, as oral rFeIFN-ω is a useful alternative management option for refractory FCGS, it may also be of particular interest in cats where corticosteroid administration is contraindicated, such as those with DM. This report underlines the relevance of rFeIFN-ω by describing two clinical cases of diabetic cats in which it was successfully administered as an alternative therapy for concurrent FCGS.

## Case presentation

### Case one

A 15 year-old castrated domestic short-hair (DSH) cat was presented to the endocrinology service of the Veterinary Teaching Hospital – Faculty of Veterinary Medicine, Techinical University of Lisbon (FMV-UTL) for polyuria/polydipsia (Pu/Pd), mild anorexia and weight loss. Prior to this consultation, the animal had been managed by the referring vet for severe dysphagia and weight loss, secondary to FCGS, diagnosed one year before. Considering the positive calicivirus status (assessed by polymerase chain reaction (PCR) analysis of an oral swab), an infectious origin had been assumed. A partial exodontia had been performed without significant improvement. Furthermore, the cat had been recurrently treated with antibiotics (cefovecin; 8 mg/kg SC every two weeks) and periodically with long-acting corticosteroids (methylprednisolone acetate; 10 mg intramuscularly every four to six weeks). On clinical examination, the cat presented with a moderate gingivitis and caudal stomatitis which extended to the palatoglossal folds. After the initial workup (hematology, biochemistry, urine analysis and abdominal ultrasound), a type II DM complicated with ketoacidosis was diagnosed. After initial stabilization with intravenous fluids and a regular insulin protocol, the cat was progressively fed with a diabetic specific diet (Purina DM) and insulin-glargine (1 IU/cat SC BID) was started. After three days of hospitalization and a good initial response to this insulin, the cat was discharged. The owners performed weekly home blood glucose curves (HBGC) and the insulin dose was adjusted according to the glycemia results. Ten weeks later, after a gradual decrease of insulin therapy, the cat went into remission of the DM and insulin therapy was stopped. However, after the remission of the diabetic clinical signs such as polyphagia, the gingivitis and caudal stomatitis got worse. Despite the good control of the DM and therapeutic trials with antibiotics (cefovecin; 8 mg/kg SC every two weeks), gastric protectants (sucralfate; 0.5 g/cat per-os (PO) BID) and NSAID (meloxicam; 0.1 mg/kg PO, SID), the FCGS progressed, with the cat developing a severe dysphagia with hypersalivation and weight loss. Due to the previous history of type II DM associated with long-acting corticosteroid therapy, an oral rFeIFN-ω protocol (0.1 MU PO SID) was started, with the owner’s informed consent. During the first two weeks of treatment, oral disinfectants (antiseptic oral solution: Collu-Hextril, Johnson & Johnson Lda; 1 diluted portion PO SID and enzymatic gel: Orozyme, Ceva; 1 cm ointment PO SID) and an antibiotic (cefovecin; 8 mg/kg SC administered once) were concurrently prescribed. After this initial therapeutic approach, only rFeIFN-ω was administered. The cat started to improve gradually, and 2 months later the owners described a significant improvement of mastication and reduced evidence of pain. The treatment was continued and animal was evaluated monthly. Six months later, at the date of the last evaluation, the cat had only a mild gingivitis and stomatitis, without significant pain. The animal had been treated only with rFeIFN-ω, which was not discontinued due to the good clinical results obtained.

### Case two

A 14 year-old castrated DSH cat was presented to the endocrinology service of the Veterinary Teaching Hospital FMV-UTL for DM monitoring. The animal had been diagnosed with DM four weeks prior to the consultation, following an acute onset of Pu/Pd and polyphagia. Apart from DM, it had been recurrently seen by the referring veterinarian due to FCGS with concurrent documented calicivirus infection, diagnosed two years previously, based on a PCR analysis of an oral swab. The cat had been intermittently treated with antibiotics (potentiated amoxicillin 15-20 mg/kg PO BID) and corticosteroids (prednisolone 0.5-1 mg/kg PO SID intermittently for three-five days) until six weeks before the development of DM. The cat was first started on veterinary lente-insulin (0.5 IU/kg BID) and fed with an appropriate diet for DM (Purina DM). At clinical presentation, the animal had a significant alveolar and caudal mucositis, with concurrent inflammation of palatoglossal folds and severe pain on mouth manipulation. No other abnormalities were observed. The owners had made some HBGC that revealed inconstant values. Considering the difficult control of glycaemia and the apparent weak response to lente-insulin, the insulin was changed to insulin-glargine (1 IU/cat SC BID). After three days of hospitalization, the animal was discharged and owners performed weekly HMBG curves. According to these measures, the insulin-glargine dose was adjusted weekly. After five weeks, the FCGS progressed with development of a severe dysphagia and hypersalivation. With the owner’s informed consent, animal was started on the oral rFeIFN-ω protocol. The use of concurrent oral disinfectants was advised but was not regularly performed by the owner. Gradually, the FCGS started to improve and the insulin-glargine requirement decreased. Eighteen weeks after starting insulin therapy, the animal achieved clinical remission of the DM with no further requirement for insulin therapy. The oral rFeIFN-ω was continued and the animal was evaluated monthly. Despite the persistence of gingivitis and caudal stomatitis, the owners reported a significant pain relief, more evident 60 days after the onset of therapy. The treatment was continued. Three months later, five months after the beginning of therapy, the cat presented with a good clinical condition with less pain on opening the mouth and a concurrent clinical improvement of the FCGS with less extensive lesions, a reduced hypersalivation and a more comfortable mastication.

## Conclusions

This report describes two cases of clinical remission of DM in cats with FCGS under insulin-glargine and dietary management. Both cases had been previously treated with corticosteroids, which are considered a risk-factor for DM in cats [10]. Although the corticosteroids were discontinued, these animals required insulin therapy and a concurrent suitable diet to control the DM. While one cat started insulin therapy with insulin-glargine, the other began the treatment with lente-insulin and was later switched to insulin-glargine. Clinical remission was obtained 10 and 18 weeks after starting insulin therapy, respectively. This is in agreement with previous studies that describe a high-rate of clinical remission in feline DM managed with insulin-glargine and suitable diets [20-22].

Concurrently, these animals were presenting with FCGS, and both cases were infected with calicivirus. This disease could have lead to a more difficult management of DM. In the first case the animal went into clinical DM remission and few days later the FCGS symptoms worsened. In the second one, the FCGS was a clinical problem during insulin therapy. Despite being associated with a previous good clinical improvement and a reduction of lesions, corticosteroids were contraindicated in both cases. Therefore, based on previous clinical trials [12], an oral protocol of rFeIFN-ω was successfully applied. In both cases, clinical improvement was gradually observed and was significantly marked (and noted by owners, who remarked that the animals started eating without discomfort and had reduced hypersalivation) around 60 days after the onset of therapy. This was particularly evident in the second case where rFeIFN-ω therapy was associated with a clinical improvement of oral lesions and a concurrent reduced insulin dose requirement which culminated in type II DM remission. This is in agreement with the previously cited work that describes an overall relief of pain in refractory cases of FCGS [12]. It is also in agreement with multiple anecdotal reports that describe a rapid improvement in well-being in cats with FCGS during oral rFeIFN-ω treatment, but with a period of three to six months being necessary in some severe cases before the lesions are fully resolved, especially where there has been regular previous use of corticosteroids (D. McGahie – personal communication). Although there are no studies that clearly detail the immunomodulatory mechanisms of oral rFeIFN-ω use, it has been proven that oral human interferon-alpha administration may potentiate a local T-helper 1 response (Th1) [23]. In fact, human interferon-alpha seems to increase the expression of gamma-interferon, a Th1 cytokine inducer, while it reduces the Interleukin-4 production, responsible for a T-helper 2 response [23]. Recognizing that the Th1 response is an important immunological pathway against viral infections [23], it seems reasonable that rFeIFN-ω and human interferon-alpha (both type I interferons) may have a similar local action. Therefore, this Th1-enhancement may explain the clinical improvement observed in these calicivirus positive cats during rFeIFN-ω therapy. Further controlled prospective studies are needed to reinforce these clinical findings, correlating them with the local immune response. These two clinical cases describe the successful use of oral rFeIFN-ω in diabetic cats with FCGS as an appropriate alternative to corticosteroid treatment where its administration is contraindicated.

## Abbreviations

BID: Twice daily; DM: Diabetes mellitus; DSH: Domestic short-hair; FCGS: Feline chronic Gingivostomatitis syndrome; FCV: Feline calicivirus (FCV); FeLV: Feline leukemia virus; FHV-1: Feline herpesvirus; FIV: Feline immunodeficiency virus; FMV-UTL: Faculty of veterinary medicine - Techinical University of Lisbon; HMBG: Home-made blood glucose; NSAID: Non-steroidal anti-inflammatory drugs; rFeIFN-ω: Recombinant feline interferon omega; Pu/Pd: Polyuria/polidipsia; PO: Per os; SC: Subcutaneous administration; SID: Once daily; Th1: T-helper 1 response.

## Competing interests

McGahie, D: employee Virbac, Carros, France. His contribution for this work was mainly as a Consultant. The other authors declare that they have no competing interests.

## Authors’ contributions

ROL, SG and MTVB carried out the clinical follow-up of both cases, making substantial contributions to the clinical management of the cats. ROL and SG were involved in data acquisition, analysis and interpretation and the drafting of the manuscript. DMcG contributed the treatment protocol. DMcG, MN and LT were involved in revising the manuscript, contributing to its intellectual content. All authors read and approved the final manuscript.

## Authors’ information

RL is a PhD fellow (FCT SFRH / BD / 62917 / 2009 Portugal) at CIISA/ Technical University of Lisbon (FMV-UTL). His current research is focussed on immune-modulation therapy in feline medicine, particularly in naturally retrovirally-infected cats. Concurrently with finalizing his PhD thesis, RL is also a clinical assistant at the Teaching Hospital (FMV-UTL), working being involved in the endocrinology service.

SG is a research assistant in the Programa Ciência 2007 (FCT-Portugal). Concurrently with her research activity, SG is also a clinical assistant at the Teaching Hospital (FMV-UTL) working in the infectious diseases service.
